# Outcomes of laser-induced thermotherapy for thyroid nodules at the West Vascular Center, Ukraine

**DOI:** 10.25122/jml-2022-0187

**Published:** 2023-01

**Authors:** Michael Ivanovich Sheremet, Oleksandr Volodimirovich Lazaruk, Oleksandr Viktorovich Shidlovskyi, Viktor Oleksandrovich Shidlovskyi, Volodimir Vasilyevich Savin, Yuriy Vasilyevich Olinik, Oksana Yuriivna Olinik, Tetyana Yuriivna Lazaruk

**Affiliations:** 1 Department of Surgery No.1, Bukovinian State Medical University, West Vascular Center, Chernivtsi, Ukraine; 2 West Vascular Center, Chernivtsi, Ukraine; 3 Department of Pathologic Anatomy, Bukovinian State Medical University, Chernivtsi, Ukraine; 4 Department of Surgery, Ivan Horbachevsky Ternopil National Medical University, Ternopil, Ukraine; 5 Department of Internal Medicine, Bukovinian State Medical University, Chernivtsi, Ukraine

**Keywords:** laser-induced thermotherapy, sclerotherapy, nodular goiter, LITT – laser-induced thermotherapy, FNA – fine needle aspiration puncture biopsy, NG – nodular goiter, TSH – thyroid-stimulating hormone TG – thyroid gland, TgAb – thyroglobulin antibody, TPOAb – thyroid peroxidase antibody

## Abstract

Thyroid nodules are common, occurring in 50–60% of healthy patients. Currently, there are no effective conservative treatment options for nodular goiter, and surgery can have limitations and potential complications. The purpose of this study was to evaluate the efficacy, tolerability, and long-term results of using sclerotherapy and laser-induced interstitial thermotherapy (LITT) to treat benign thyroid nodules. A retrospective analysis was conducted on 456 patients with benign nodular goiter who received LITT. The volume of the nodular goiter was measured at 1, 3, 6, and 12 months post-treatment, and a repeated fine needle aspiration (FNA) with the cytological examination was performed to verify the structure of the nodular goiter in the long term. The results showed that LITT was an effective method for treating nodular mass (nodules), as evidenced by a decrease in the volume of NG by 51–85% after 6–12 months. FNA results 2–3 years after LITT showed no thyrocytes, only connective tissue, indicating the efficacy of LITT for benign thyroid nodules. LITT is highly effective in most cases, often resulting in the disappearance or significant decrease in nodular formations.

## INTRODUCTION

Thyroid nodules are a common occurrence in clinical practice, with a prevalence ranging from 3–7% based on physical examination and up to 76% based on thyroid sonography [1–7]. Although most thyroid nodules, according to fine-needle aspiration puncture biopsy, are benign, do not cause compression complaints, and do not require specific treatment, individual nodules can increase in size, causing symptoms or neck deformation in the patient, and can lead to excessive production of thyroid hormones [8–11]. Therefore, some patients with benign thyroid nodules require corrective treatment.

According to recent research, an alternative to surgical treatment of benign thyroid nodules is laser-induced thermotherapy (LITT) of thyroid nodules under ultrasound control [12–15]. LITT of thyroid nodules was developed and introduced in 1998 to treat solid thyroid nodules [14–16].

The LITT is a minimally invasive treatment method based on the combined action of light and temperature at the end of the light pipe that is brought into the tumor itself (node). A temperature of 41–46 degrees is applied, affecting the protein structures of the cell without destroying it as a whole, but further cell division of the nodule parenchyma becomes impossible [17–20]. LITT is widely used in countries such as America, Western Europe, Asia, and Japan [21–25].

According to current guidelines for treating thyroid nodules, ultrasound-guided laser-induced thermotherapy of thyroid nodules can be used to treat solid and cystic nodules. The thyroid gland, gradually increasing in size, causes symptoms of thyrotoxicosis or creates cosmetic issues [22–27].

The objective of this study was to evaluate the efficiency, tolerability, and long-term results of the use of sclerotherapy and LITT for the treatment of benign thyroid nodules.

## MATERIAL AND METHODS

The Lahta Milon^®^ diode laser 1060/90 model was used. The LITT was conducted according to the following parameters: wavelength 1060 NG, continuous mode, and radiation power ranging from 2.5 to 3.2 W. A 400 µm quartz laser fiber with a torsional end was used, which was inserted into the needle lumen of a 5 mm fiber coming out of the needle. After LITT, changes in the node and perinodal tissue were determined on the second day, in 2 weeks, in 1, 3, 6, 9, and 12 months after the manipulation. Changes in the volume of nodes and their structure were studied using the TOSHIBA Aplio XG A ultrasonic device. Thyroid function was determined one month after the LITT.

The inclusion criteria for this retrospective study were as follows:

- Solid nodules from 0.5 to 45 cm^3^ (fluid component ≤10%); if the fluid component percentage was higher, fluid component evacuation and chemical sclerotherapy with 96% ethanol were performed;
- Diagnosis of benignity by 3–4 fine needle aspiration biopsies – Bethesda Category II, 2017;
- Normal levels of TSH (thyroid-stimulating hormone), FT4 (free thyroxine), TgAb (thyroglobulin antibodies), TPOAb (thyroid peroxidase antibodies), and calcitonin
- The presence of local compression symptoms;
- Refusal of an open operation or the availability of contraindications to it.

Informed consent was obtained in writing from all patients meeting the inclusion criteria. From December 2019 to June 2022, 456 patients were treated. The distribution of patients by age, gender, and type of nodes is presented in Table 1.

**Table 1:** Patient characteristics.

Number	456
**Age**	18–78 (56)
**Gender – female/male**	399/57
**Neoplasm type:**	
1. Adenomatous nodes	56
2. Colloid goiter	237
3. Nodular goiter with cystic degeneration	163

## RESULTS

Patients were positioned lying on their backs with their heads thrown back and a pad under the shoulder blades. The laser light guide was inserted from the side of the isthmus into the lateral part of the node. The input of the light guide was visualized under ultrasound navigation, while the zone of the triangle, including the reverse laryngeal nerve and/or esophagus, accounted for the minimum amount of thermal energy. To avoid damage to adjacent organs, we maintained a distance of at least 15 mm from the fiber tip to the distal edge of the nodule and a 10 mm distance from the surrounding structure and the edge of the nodule capsule. Local anesthesia with 2% lidocaine hydrochloride solution was applied at the site of the light guide insertion, while sedation was not used since we used the manifestation of a minor pain symptom or burning sensation as a guideline for the final cessation or reduction of energy delivery.

A positive result was defined as a reduction of ≥50% in 1 year after treatment. Changes in the node, perinodal tissue, and gland function after LITT were determined on the second day, 2 weeks later, 1, 3, 6, 9, and 12 months after the manipulation.

On the second day after the LITT, the volume of nodes increased by 20–35% compared with the initial data. The percentage of their growth depended on echogenicity. Hyperechogenic nodes increased by 20–25%, isoechoic – by 25–30%, and hypoechoic – by 25–35%. An increase in the volume of nodes is a consequence of aseptic inflammation and swelling of the tissue inside the node.

**Figure 1:**
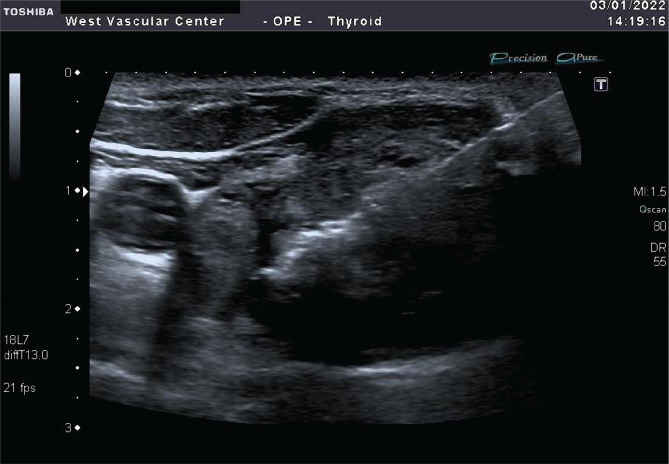
Laser fiber in the center of the node.

Table 2 shows the distribution of significant reductions in nodule size following LITT. This table shows that among the nodes of up to 1 cm^3^ after the LITT, the entire part decreased by more than 75%, 48 (37.4%) nodes decreased by more than 75%, and 84 (63.6%) nodes disappeared completely. Among the nodules with 1–2 cm^3^ volume, all nodules decreased by more than 75%, of which 151 (75%) or more and 62 (29.1%) – scar tissue with zero volume remained. At the same time, among nodular formations from 2 to 4 cm^3^, there was not a single node that disappeared completely. However, in 60 (89.5%) patients, the node volume decreased by more than 75%, and in 7 (10.5%) – from 50% to 75%. When analyzing the reduction of nodes with a volume from 8 to 45 cm^3^, it should be emphasized that all formations probably decreased after sclerotherapy with ethanol and after using the LITT in more than 4 projections. This technology made it possible to fully heat the entire tissue of the node with a laser (Figure 1).

**Table 2:** Relative decrease in the volume of thyroid nodules 1 year after LITT.

Decrease after LITT	to 1 cm^3^ n=132	from 1 to 2 cm^3^ n=213	from 2 to 4 cm^3^ n=67	from 4 to 8 cm^3^ n=25	from 8 to 45 cm^3^ n=19
**Completely disappeared**	84/100%	62/100%	-	-	-
**75% and more**	48/74.6%	151/70.9%	60/89.5%	13/52%	7/37%
**50–75%**	-	-	7/10.5%	6/24%	5/26%
**40–50%**	-	-	-	6/24%	5/26%
**Less than 40%**	-	-	-	-	4/11%

When examining patients after 1 year, the positive therapeutic effect was maintained. Thus, most nodes decreased by more than 40–50% (Table 2).

We analyzed the LITT efficiency depending on the number of projections and the duration of laser exposure to the node tissue. By the number of projections of laser exposure, we understand not just the movement of the laser light guide but the number of punctures of the puncture needle with re-insertion of the light guide after removing carbon deposits from it. At the same time, direct proportional dependence between the number of projections and a decrease in the volume of thyroid gland nodes was established.

**Figure 2:**
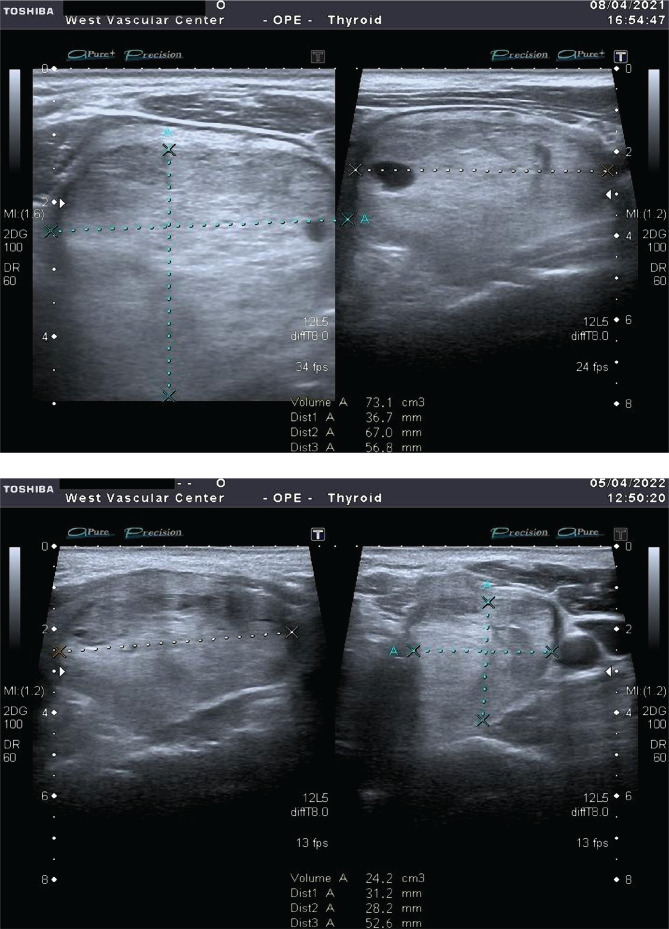

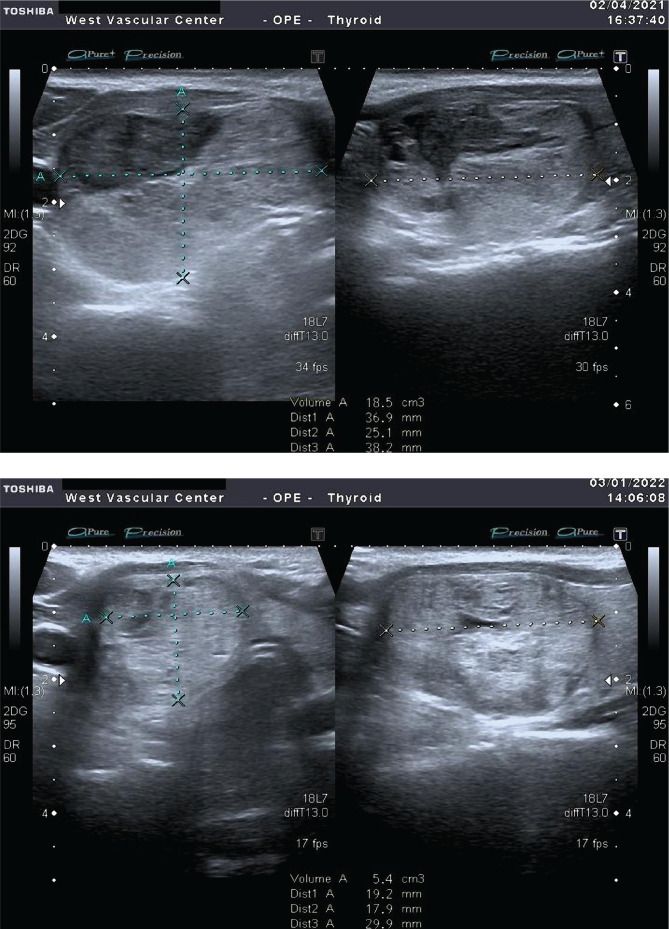
Node volume before and 12 months after LITT.

In 15 out of 44 (34.1%) patients with "large nodes" from 8 to 45 cm^3^ after the first LITT session, the efficiency was observed in 25 to 42%. Subsequently, a second LITT session was performed, and in 6 (13.6%) patients, two LITT sessions were performed. As a result of the sessions, the volume of nodes decreased by more than 55%, while the compression syndrome significantly decreased and the need for the surgery vanished (Figure 2).

Transient sub-febrile fever and local pain were reported in some patients, but the absolute number and percentage of patients reporting these side effects were small [sub-febrile fever: 3 patients (0.6%); pain: 2 patients (0.2%)].

According to repeat fine-needle aspiration biopsies (FNA) taken 2–3 years after LITT, the microscopic changes that occurred in the treated node were determined. The results showed that the ablation zone was surrounded by a peripheral rim of dense fibrous tissue. The area of destruction was characterized by the presence of amorphous material, macrophages, multinucleated giant cells, and lymphocytes, but no thyroid cells. Viable tissue was visible at the periphery, separated from the ablation zone by fibrous tissue.

The ultrasound characteristics of the node were represented by cicatricial, fibrous changes with hyperechoic areas that give a positive echo + shadow and hypoechoic areas of irregular shape as a result of proliferative and regenerative processes in the node.

## DISCUSSION

Recent studies have proposed a unified terminology for laser-induced thermotherapy (LITT) of thyroid nodules using ultrasound guidance, in which nodules are classified as "small" if they have a volume of ≤10 cm^3^, "medium" if the volume is 11–30 cm^3^, and "large" if the volume >30 cm^3^ [28]. In our study group of 15 patients, the average size of the thyroid nodules was 38 cm^3^ with a range of 20–45 cm^3^, which classifies them as "large" according to recent proposals for the classification of LITT under sonographic guidance. These large nodules required a significant ablation volume for treatment.

Another group of researchers believes that during LITT, an increase in the photocoagulation volume in the transverse direction to the direction of needle insertion can be achieved by simultaneous illumination of more than one optical fiber located at a distance of 10–15 mm [28]. In addition, according to their data, an increase in the volume of ablation in the direction of needle insertion can be achieved by a maneuver of retraction and re-entry of the optical fiber in more than 6–8 projections. Using these approaches in combination with repeated LITT sessions has given experts very good technical results in reducing large nodules by 75% after 1 year. The effect of the treatment was long-lasting; the long-term effect lasted from 2 to 3 years after the initial treatment. The research showed that to obtain good results, an energy dose exceeding 500 J/cm^3^ of nodal tissue is required [29, 30]. In this series of patients, the average amount of energy delivered to the nodes was 461 J/cm^3^ of tissue. However, for very bulky nodules, it was necessary to add slightly less energy per 1 cm^3^; otherwise, it would lead to significant burns, and the treatment duration would be long enough, which is not acceptable for the treatment of such patients.

In our treatment series, patients tolerated laser ablation well and experienced minimal complications. Some patients had temporary fever and pain, but the number of patients affected was small (0.6% for fever and 0.2% for pain). These percentages align with findings from previous studies [31]. However, there is limited data from randomized controlled trials comparing laser ablation to other ultrasound-guided techniques.

One study that compared LITT to radiofrequency ablation, another minimally invasive treatment for thyroid nodules, was conducted on 58 patients with solid thyroid tumors. The study found that while radiofrequency ablation resulted in a greater reduction in nodule mass 12 months after treatment, both LITT and radiofrequency ablation had similar technical success rates and statistically significant improvements in compression symptoms and symptom scores [27]. Additionally, a retrospective analysis of 320 thyroid nodules in 320 predisposed patients compared laser ablation to microwave ablation. The study found that for nodules larger than 13 mL, the results of laser interstitial thermotherapy at 6 months and later were stronger than those obtained with microwave ablation, while overall group characteristics were comparable between RF and LITT [29].

R. Valcavi [16] and C. M. Pacella [32] observed hematoma in 2% of patients in the postoperative period, which was asymptomatic and was detected only by ultrasound in the form of a hypoechoic layer around the thyroid gland. No surgical intervention was required. The hematoma was resolved by the end of the third week. Irritation of the vagus nerve in the form of bradycardia was observed in 2%. Another 2% of patients complained of coughing during the procedure. 2% of patients complained of dysphonia. Laryngoscopy showed limited mobility of the vocal fold. However, after 2 months, the function of the vocal folds resumed. Rare side effects include skin burn (0.3%) and stridor (0.3%). Thyroid gland dysfunction was observed in 3% of patients (1 patient had hyperthyroidism, 1 – had hypothyroidism) [30].

Summing up the data obtained, the main advantages of the LITT over surgery should be noted: 1) it is a minimally invasive technology that has the same clinical results as surgery; 2) the LITT can be performed under local anesthesia and on the outpatient basis; 3) intra- and postoperative complications after the LITT are extremely rare and easily acquired; 4) the dynamic LITT technique allows overcoming the preliminary limiting factors of the technique, such as large node sizes, multinodular formations. Finally, the LITT is possible in most cases in patients with benign thyroid nodules.

## CONCLUSION

The technique of laser-induced interstitial thermotherapy of benign thyroid nodules is highly effective since it leads either to the disappearance or to a significant decrease in nodular formations in the vast majority of cases.

The greatest efficiency of this technique is manifested in the treatment of small nodular formations – up to 10 cm^3^. However, nodes of this size are most common and, with a significant increase in the future, can have an adverse effect on the quality of life associated with their progressive growth and, as a result, the occurrence of a compression syndrome, which may require surgical treatment.

Also, the laser-induced interstitial thermotherapy saves the parenchyma of the gland and avoids the development of hypothyroidism and the LITT technique of colloid nodes with cystic transformation is safe and effective and based on it, we can recommend this technique for wide use in medical practice.
